# mHealth as a Key Component of a New Model of Primary Care for Older Adults

**DOI:** 10.2196/82262

**Published:** 2025-12-19

**Authors:** Jean Woo, Ruby Yu, Maggie Wong, Ken Cheung, Nicole Fung

**Affiliations:** 1 Department of Medicine and Therapeutics, Faculty of Medicine, Chinese University of Hong Kong, 9/F, Lui Che Woo Clinical Sciences Building, Prince of Wales Hospital, Shatin, NT, China (Hong Kong), 852 35053493, 852 26373852; 2 Jockey Club Institute of Ageing, Chinese University of Hong Kong, Shatin, China (Hong Kong)

**Keywords:** intrinsic capacity, life expectancy, health span, integrated care of older persons, healthy aging, primary care

## Abstract

With population aging, an increase in total life expectancy at birth (TLE) should ideally be accompanied by an equal increase in health span (HS), or by a trend in increasing HS/TLE ratio. Hong Kong has one of the longest life expectancies in the world; however, there is a trend of declining HS/TLE ratio, such that the absolute number of people with dependencies is increasing. To address this challenge, the World Health Organization proposed the model of integrated care for older people (ICOPE) that combines both health and social elements in community care, using the measurement of intrinsic capacity (IC) as a metric for monitoring the performance in different countries. The use of technology is essential in achieving a wide coverage of the population in assessing IC, followed by an individually tailored plan of action. This model can be adapted to different health and social care systems in different countries. Hong Kong has an extensive network of community centers, where the basic assessment may be based, followed by further assessments and personalized activities, and referral to medical professionals may only be needed in the presence of disease. Conversely, the medical sector may refer patients to the community for activities designed to optimize the various domains of IC. Such a model of care has the potential to address manpower shortage and mitigate inequalities in healthy aging, as well as enable the monitoring of physiological systems in community-dwelling adults using digital biomarkers as a metric of IC.

## Demographic Shift Resulting in Increasing Dependency

As in many developed economies, Hong Kong is experiencing a rapid demographic shift, with its aging population growing at an unprecedented rate [1]. As of 2021, approximately 20% of Hong Kong’s population was aged 65 years or above, and this figure is projected to rise to over 35% by 2046 [2]. This trend poses significant challenges to the health care system, elder care services, and informal caregivers, making it increasingly difficult to meet the complex health and social needs of older adults. As a result, an increase in health span has not matched the pace of increase in total life expectancy, resulting in an increase in absolute numbers of people with dependency needs [3].

## The Urgency of the Problem

In spite of the existence of an excellent health system that is low-cost and free to those in poverty, a system that should theoretically mitigate health inequalities in the context of extreme wealth inequality, this dependency burden has resulted in health inequality due to unequal access [4]. Resources—such as clinical screenings, rehabilitation, and caregiver support—remain limited with many barriers to access. Many older adults, particularly those with mobility constraints or financial difficulties, struggle to access timely and appropriate care. The burden also extends to family caregivers, who often lack adequate support and knowledge, leading to increased stress and burnout [5]. At the same time, training programs in skills to manage age-related health conditions of personnel in elder care are not fit for purpose, and the number of such staff is not sufficient. Health and social care systems are also fragmented, such that early signs of functional decline may go unnoticed, leading to preventable complications and hospitalizations.

## Need for Integration of Health Social Care Using a Stepped-Care Model, Shifting From Hospital to Community, From Sickness to Prevention

To address these challenges, a structured and multitiered approach is essential. The stepped-care model provides a framework where different levels of intervention are applied based on the severity of a person’s needs. This aligns with the Integrated Care for Older People (ICOPE) model, developed by the World Health Organization (WHO), which emphasizes early detection, prevention, and community-based support to promote healthy aging and independent living [6]. The ICOPE model focuses on monitoring key domains such as mobility, cognition, psychological well-being, nutrition, vision, and hearing to identify risks and implement timely interventions [7], in contrast to conventional healthcare that prioritizes diagnosing and managing individual diseases in isolation. Monitoring and managing declines in the domains of intrinsic capacity (IC), such as hearing, vision, memory, and mobility, can greatly affect quality of life and overall health [8]. By prioritizing prevention and proactive care, this innovative framework encourages a shift from reactive disease treatment to a proactive, function-based approach, empowering older adults to take charge of their health and well-being [9]. Preventive strategies for geriatric syndromes can significantly impact adverse health outcomes and dependency, as these syndromes increase hospital and outpatient utilizations [4].

Building upon IC as a monitoring tool for healthy aging, the ICOPE model supports the optimization of IC and functional ability of older adults, addressing their social and health needs in a holistic and person-centered approach [10]. The care pathway consists of four steps for delivering integrated care: basic assessment, in-depth assessment, development of a personalized care plan, implementation, and monitoring.

## Technology Has a Key Role in the New Model of Primary Care in Raising Health Literacy

Technology has an important role in this care model, in enabling population-wide coverage. The concept of mHealth as a crucial tool for achieving healthy aging has been promoted by the WHO, particularly through its mAgeing program, as part of its global strategy to support older adults in maintaining their IC and functional ability [11]. Increasing adoption of technology and smartphones among older adults presents an opportunity to enhance health care accessibility through digital solutions. For example, smartphone adoption among Hong Kong’s older adults rose significantly from 68% in 2020 to 90% in 2022 [12]. Digital tools can bridge the gap in service accessibility, providing self-management support, health education, and real-time resources that empower both older adults and caregivers.

Currently, various barriers exist for the successful implementation of the ICOPE care pathway in primary care. These include the lack of active engagement of stakeholders from community health and social care sectors and ageism, regarding age-related changes and declines in intrinsic capacity as an inevitable accompaniment of aging, focus on chronic disease and not taking into account geriatric syndromes, and fragmentation of care delivery systems into medical, and social compartments, hospitals and community settings, and absence of funding mechanism [13].

## The Hong Kong Research Initiative in Using Technology as a Key Component of Providing a New Model of Integrated Health and Social Care in the Primary Care Setting

In order to address the unmet needs of Hong Kong’s aging population and to promote equitable healthy aging, a project was initiated to raise health literacy relating to prevention, assessment, and management of IC declines and geriatric syndromes, among older adults, informal and formal caregivers, using information technology to create a mobile application covering assessment, and available neighborhood services (Multimedia Appendix 1). It is linked to an extensive series of topics focusing on commonly encountered problems, the underlying cause, and evidence-based management strategies. The design and development of the mobile application included repeated feedback from potential users: older adults themselves, informal and formal carers, through user testing sessions and focus group discussions. Specific age-friendly features were incorporated to facilitate adoption among older adults (Table 1).

The iHealth Screen mobile app integrates three key modules to support the ICOPE care pathway: (1) a health screening module covering IC and other 11 geriatric tests such as sarcopenia, cognition, and nutrition; (2) a self-care education hub offering credible content developed by geriatrician and nurses; and (3) a GPS-enabled community resource repository that connects users to 28 categories of medical and social care services across Hong Kong. These services include community health services, rehabilitation support, assistive tool rentals, barrier-free transportation, and carer support services, among others.

**Table 1.** Age-friendly design of iHealth Screen.

Key aspects	Age-friendly features
Font size	Enable standard and large font size options to suit for individual needs
Buttons and icons	Utilize large buttons and easily recognizable icons—such as a muscle icon for sarcopenia test and a brain icon for cognitive function tests— to facilitate older adults’ comprehension
Color contrast	Use of high-contrast color, such as dark black text on a light yellow-orange background for clear visibility
Navigation	A chatbot-style interface guides users through health tests and community resource searches using predefined options. This design resembles familiar messaging and daily-use apps, eliminating the need for manual text input
Voice instruction	Voice-over instructions with text and images for particular health tests requiring additional steps, such as sarcopenia and cognitive tests, to support users who may benefit from auditory explanations
In-app onboarding	Offers welcome guidance and interactive walkthroughs to assist first-time users in familiarizing with the app’s features and navigation

The health screening module enables users to complete assessments and receive instant results along with tailored educational recommendations. It incorporates standardized questionnaires and an embedded algorithm that processes predefined user responses to generate results and guidance. For instance, intrinsic capacity is assessed using a validated local questionnaire [14] that measures five domains: cognitive capacity, locomotor capacity, vitality, psychological capacity, and sensory capacity. The results are presented as a composite score ranging from 0 (no impairment) to 5 (impairment in all domains), and are categorized into three levels: 0 (robust), 1‐2 (mild impairment), and 3‐5 (severe impairment). Impaired domains are clearly highlighted to prompt user attention and guide follow-up actions. Users can also review past results and access an integrated health report to monitor changes over time and support early detection and intervention.

The mobile app is freely accessible to the public on both Android and iOS platforms. To ensure ease of use, individual users are not required to create personal accounts and can access all features directly. Upon first use, users are invited to complete a brief, optional demographic survey, including age group, gender, education level, and self-identified role. Each user is assigned a unique, anonymized ID for backend tracking of app usage, without collecting names or sensitive personal data. All data is securely stored on the university’s web hosting infrastructure, ensuring security measures to prevent unauthorized access or data breaches. A comprehensive privacy policy is published within the app to inform users of their rights and ensure compliance with ethical and legal standards.

In addition to individual use, the app offers an institutional interface designed for potential community partners to integrate ICOPE care into routine operations. This module allows organizations to create accounts for their members, monitor health conditions, and plan targeted healthy aging initiatives. For institutional users, informed consent is obtained prior to collecting any personal information beyond the standard anonymous data. This ensures that all data collection is conducted transparently and ethically, with explicit user permission.

In particular, the usefulness of the mobile application was explored in various community centers, which provide various social support services as well as group activities. These are run by nongovernment organizations (NGOs) covering most areas of Hong Kong, with medical, nursing, and allied health input to varying extents. To adapt to local needs, where many foreign domestic helpers are employed to help with the care of older adults, translations into Tagalog, Indonesian, and Putonghua have been incorporated into the educational resources. An indicator of the perceived usefulness of this tool is provided by the following user statistics between November 2024 to March 2025: 7000 downloads of the app from app distribution platforms; 3300 users completed IC and related tests; and 2000 community resource searches.

1820 users completed 4626 IC, frailty, sarcopenia, and nutrition; and 2177 community resources searches by 1198 users, with the most frequently accessed being nutrition consultation, carer support service, followed by rehabilitation, assistive tool rental, and fall prevention. Qualitative feedback from older adults and caregivers highlighted that the app provides an accessible and consistent self-care tool for monitoring health and accessing resources, challenging the assumption that older individuals cannot use mHealth tools independently.

Despite positive engagement indicators, sustained use of the app remains a challenge. Limited awareness of health management often reduces motivation for regular app usage, as the need for health assessments is not always perceived as necessary. The value of standardized tools for monitoring intrinsic capacity and geriatric health is also not widely understood, with a preference for practical health information and demonstrations over completing assessments. Community outreach has been identified as an effective strategy to improve mHealth adoption, emphasizing the need for educational initiatives that not only focus on the technical aspects of app usage but also highlight the value of healthy aging and proactive health monitoring.

The creation of this product provides a tool that may be used as a key component of a new model of integrated primary care for older people, building on existing health and social care infrastructure. Its use promotes empowerment for older adults and their carers, self-management, raising health literacy related to aging matters, participation in health-promoting group activities, linkage to existing medical and social services, other community resources, as well as reduces carer stress. The importance of raising health literacy cannot be emphasized enough, as one of the most significant issues in enabling health inclusivity for as wide a population as possible. It has been shown that a modest increase in health literacy may deliver significant health gains that can be translated into increasing a country’s GDP as well as reducing health care costs [15]. This is supported by a recent literature review showing that health literacy combined with planned physical activity can increase self-efficacy, cognitive function, quality of life, and reduce frailty scores, falls risk, and depression scores [16]. However, providers need to ensure that technology does not create new barriers for older people to access information and services. In the development of this digital tool, great care has been placed in the design precisely to minimize this risk.

Creation of this digital tool enables the effective implementation of the ICOPE model for healthy aging in Hong Kong, following the principles of the model. The ICOPE model articulates the principles: countries have different economic developments, health and social care systems, so that the actual model may vary. In the case of Hong Kong, the tool may form an important link between the medical systems (hospitals, outpatient clinics) with the large network of social centers (eg, NGOs), based on the principles of social prescribing (Multimedia Appendix 2). This tool could be used as a first step to determine needs, followed by actions that may largely take place in NGO centers in terms of educational programs, more detailed functional assessments, and regular participation in group activities such as frailty prevention programs. The advantage of the app goes beyond the provision of just another tool, in its potential in countering ageism by raising health literacy relating to management of geriatric syndromes and the aging process itself, and also facilitates stakeholder engagement by offering a complementary pathway to continuing community care. The establishment of this new model of care has the potential to address the lack of manpower, in addition to mitigating inequalities in healthy aging. It may also enable the monitoring of physiological systems in community-dwelling adults using digital biomarkers, a trend that is transforming health care and personal health management [17].

## Supplementary material

10.2196/82262 Multimedia Appendix 1 iHealth Screen.

10.2196/82262 Multimedia Appendix 2 A new model of care.

## Abbreviations

HS: health span
IC: intrinsic capacity
ICOPE: integrated care for older people
NGO: nongovernment organizations
TLE: total life expectancy at birth
WHO: World Health Organization
