# Supplementary figures and images for: mHealth as a Key Component of a New Model of Primary Care for Older Adults
Source: JMIR Form Res. 2025 Dec 19;9:e82262. doi: 10.2196/82262 (PMC12716121; doi:10.2196/82262)

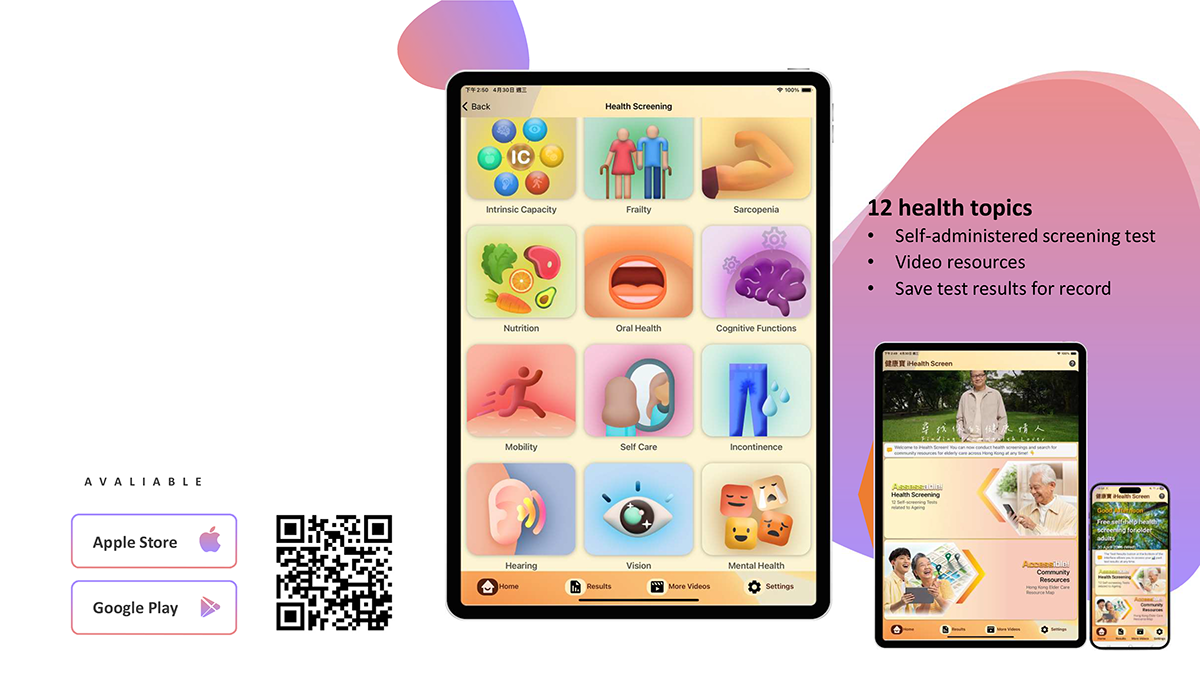

Supplement: Multimedia Appendix 1 [file formative-v9-e82262-s001.png]

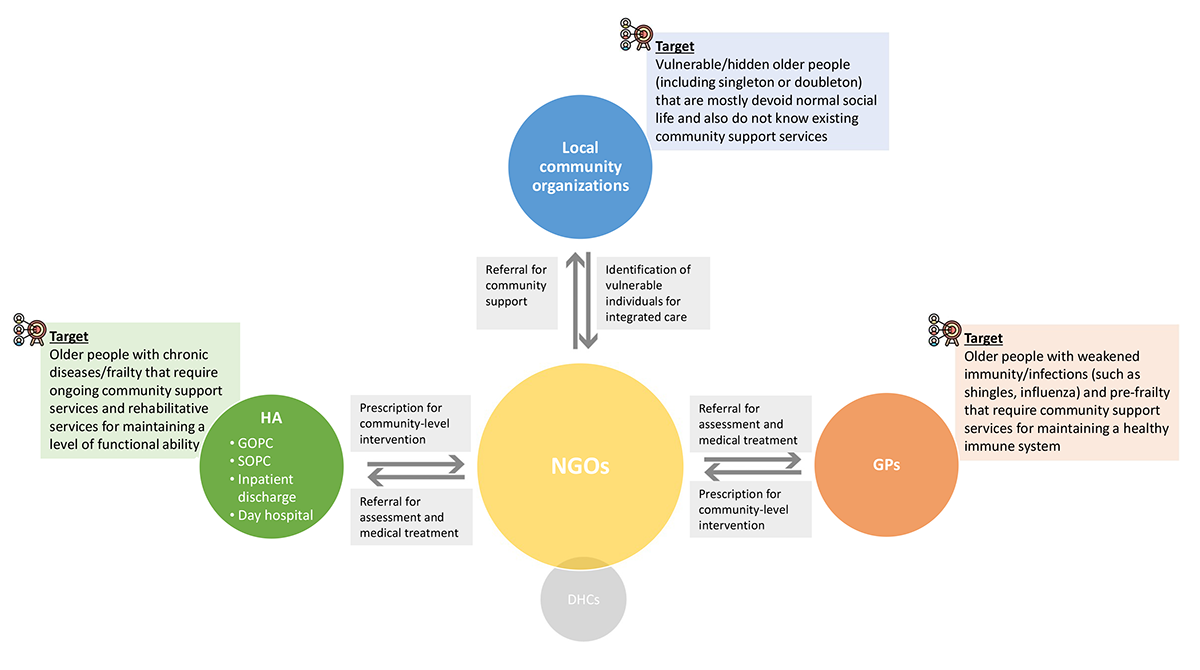

Supplement: Multimedia Appendix 2 [file formative-v9-e82262-s002.png]
